# Predicting future forest fire occurrence probability based on drought characteristics at various temporal scales in P. R. China

**DOI:** 10.1371/journal.pone.0337473

**Published:** 2025-12-02

**Authors:** Xianzhuang Shao, Chunlin Li, Yu Chang, Zaiping Xiong, Hongwei Chen, Rongping Li

**Affiliations:** 1 Key Laboratory of Forest Ecology and Silviculture, Institute of Applied Ecology, Chinese Academy of Sciences, Shenyang, P. R. China; 2 Colledge of Geography and Environment, Shandong Normal University, Jinan, P. R. China; 3 School of Life Sciences and Engineering, Shenyang University, Shenyang, P. R. China; 4 Institute of Atmospheric Environment, China Meteorological Administration, Shenyang, P. R. China; Euro-Mediterranean Center for Climate Change: Fondazione Centro Euro-Mediterraneo sui Cambiamenti Climatici, ITALY

## Abstract

Future climate change will lead to extreme weather events, such as droughts, which may exacerbate forest fire regimes. However, the impact of future drought characteristics on forest fire regimes has rarely been reported in China. Here, we employed principal component analysis to reduce the dimensionality of drought characteristics, and then used geographically weighted logistic regression models to develop predictive models. These models were applied to future climate simulations under different scenarios to provide projections for different periods, which were then compared with the historical period (2000−2019) to assess the relative changes. We found that the model performed well in its predictions (AUC > 0.75). By comparing the Brier scores, it was found that the models with better predictive performance were those using the SPEI-1 and SPEI-12 timescales. We also found that in the near and medium term of the future, with climate change, the forest fire occurrence probability in most forest land of northern China (NWC, NC, and NEC), especially in Northeast China (NEC), shows an increasing trend, but a decreasing trend in most forest land of southern China (SC, SWC, and EC). Our research can provide a scientific basis for the development of future forest fire management practices that mitigate drought stress according to local conditions.

## 1 Introduction

Climate models project that future drought conditions vary across regions and climate change scenarios [1]. In China, projections show that continuous dryness will occur in the southwestern region, while wetness in the northwestern area under all the new SSP-RCP scenarios [2]. These changing drought conditions may have profound impacts on forest fire regimes. Forest fire regimes are a set of parameters that characterize forest fires, mainly including the frequency/probability, seasonality, severity, spatial pattern of forest fires, and the size of burned area [3,4]. The forest fire occurrence probability is an important parameter of forest fire regimes. How forest fire occurrence probability responds to droughts is of significant concern. A deeper understanding of the relationship between drought and forest fire occurrence probability under future climate change conditions can provide a scientific basis for taking climate change adaptation measures [5], making forest fire management decisions [6,7], and reducing fire losses [8].

Several drought indices have been used for drought monitoring, such as the Palmer Drought Index (PDSI) [9], Standard Precipitation Index (SPI) [10], Standard Precipitation Evapotranspiration Index (SPEI) [11], and Standard Precipitation Drought Index (SPDI) [12]. Unlike the Palmer Drought Severity Index (PDSI), which has a fixed time scale, the SPEI has a temporal multi-scalar property [13]. The SPI index is based solely on precipitation data, and compared to the Standardized Precipitation Index (SPI), the SPEI combines temperature and precipitation data [14]. Out of these indices, the SPEI index is characterized by multiple temporal scales and non-stationarity [15,16] and takes into account temperature as well as potential evapotranspiration, which is more suitable for monitoring droughts in the context of a warming climate [17,18]. A drought event can be characterized in terms of drought frequency, drought duration, and drought intensity [2].

Many studies have shown that the frequency [19,20], duration, and severity of droughts will increase under future climate change conditions [21]. For example, the area of the globe experiencing extreme droughts will increase to 7% by the end of the century from 3% during 1976–2005 [22]. There will be an increase in drought duration and intensity in the northeastern and northwestern regions of China [23]. However, how the forest fire regime responds to these changing drought characteristics is seldom documented. Current studies mainly relate forest fire regimes to drought indices, not drought characteristics. For example, the number of forest fire occurrences in Mexico is negatively correlated with SPEI [24]; the number of large fires and burned area in the western U.S. have a certain correlation with the Palmer Drought Index (PDSI) [25]; Russo et al. (2017) found that drought and forest fires were correlated at the local scale [13]. The relationship between forest fire occurrences and drought indices could be used to develop empirical models to predict future forest fire regimes [26,27], such as Turco et al. (2018b) showed that warmer and drier conditions due to climate change increased burned area in the European Mediterranean region by constructing a regression model of burned area versus SPEI [28]. 97% of mega-fires occurred during the heat wave period in Portugal from 1981–2010 [29]. In China, researchers employ methods such as the Random Forest algorithm [30] and extreme gradient boosting models [31] to forecast future forest fire regimes based on factors including vegetation, climate, and human activities. Some scholars have also employed a Bayesian hierarchical modeling framework to quantify the impact of drought on the frequency of wildfires in China’s subtropical forests [32]. Research in China predominantly employs machine learning models to forecast future fire regimes, with limited consideration given to spatial non-stationarity and infrequent inclusion of drought characteristics as an influencing factor within these models. In summary, the majority of these studies in China and other countries did not consider the spatial non-stationarity between forest fire regimes and drought characteristics [32]. Based on prior research, we employ run theory to identify drought characteristics and establish the geographically weighted logistic regression model for predicting forest fire occurrence probability in China based on these drought characteristics to address gaps in existing literature, which rarely incorporate drought characteristics as influencing factors and seldom account for their spatial non-stationarity.

In this paper, we calculated SPEI using future climate variables (temperature, precipitation) from the future scenario CMIP6 and identified drought characteristics at four temporal scales (SPEI-1, SPEI-3, SPEI-6, and SPEI-12) from the SPEI data using run theory. First, we transformed the drought characteristics using principal component analysis to address multicollinearity issues. Then, the global logistic regression model and a geographically weighted logistic regression model based on the drought characteristics, along with natural and anthropogenic factors, were constructed. Finally, the spatial and temporal pattern of forest fire occurrence probability in China was predicted for the near-term (2022–2040), medium-term (2041–2060), and long-term (2080–2099) periods using the multimodal mean ensemble (MME). Our results could provide a basis for the development of future forest fire prevention and control measures in China.

## 2 Materials and methods

### 2.1 Study area

**Fig 1:**
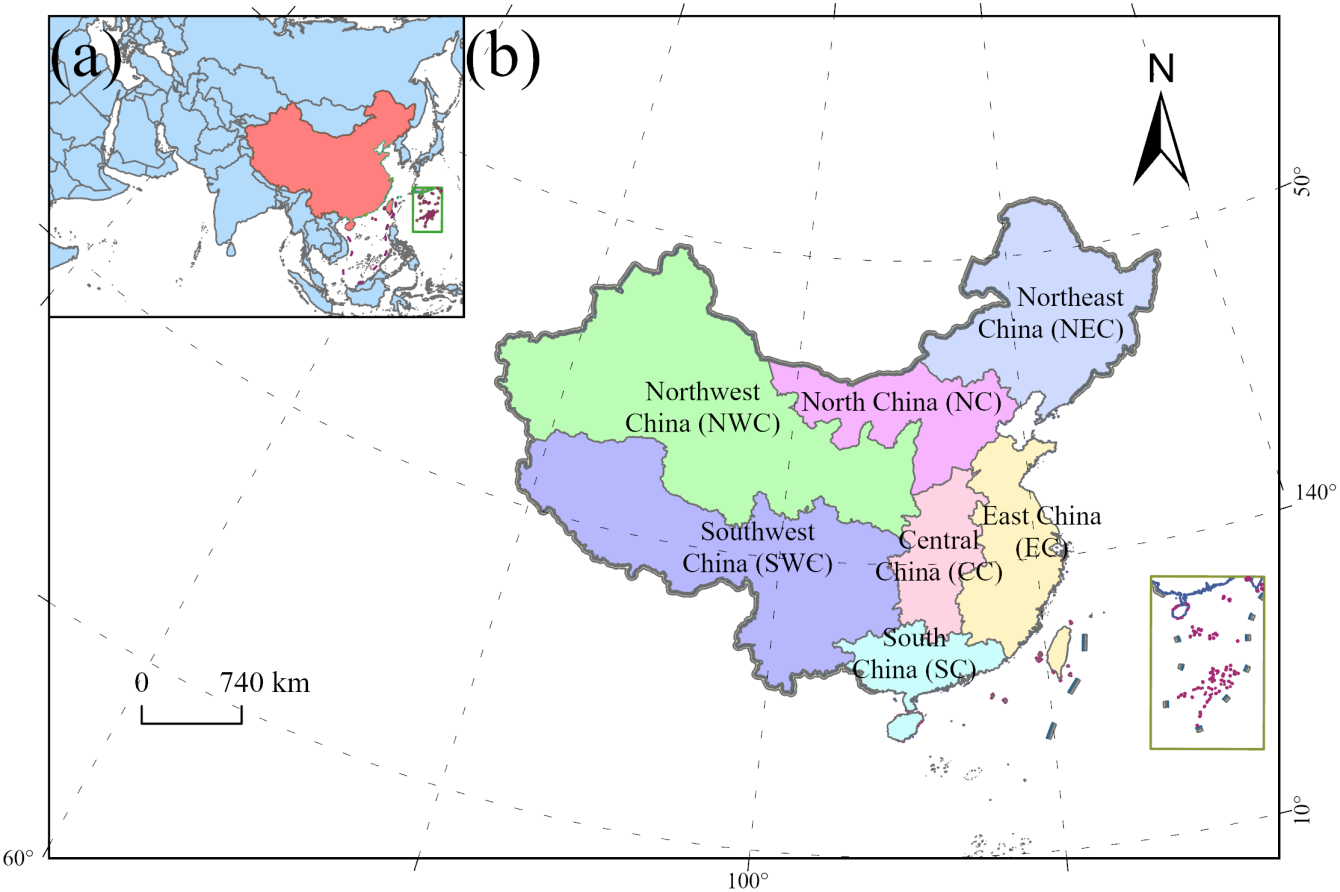
The seven geographical divisions of China(b) and China’s location within Asia(a). The map is produced based on the standard map with review number GS (2023)2767 downloaded from the website of the Standard Map Service of the State Administration of Surveying of the People’s Republic of China, Mapping and Geoinformation(http://bzdt.ch.mnr.gov.cn/), with no modification to the base map.

China is located in eastern Asia, on the western coast of the North Pacific Ocean. Its climate is influenced by the monsoon, resulting in significant differences in precipitation patterns both in time and space [33]. Due to China’s large population, diverse climatic conditions, and diverse vegetation types, there are large regional differences in the spatial and temporal distribution characteristics of forest fires. For example, the burned area in northern China is larger than that in the south, but the frequency is lower than in the south [34]. China’s meteorological drought events are of long duration, wide range, and high severity, and are spatially concentrated in the central and western regions [35]. In areas with high vegetation cover, high temperatures cause vegetation to dry out and become more flammable [36]. Forest fires occur significantly more frequently in the eastern coastal regions than in the western inland areas [36]. Forest fires frequently occur in moderately moist regions, where dryness levels are sufficiently high relative to local moisture conditions to permit changes in the combustibility of local fuels [34].In subtropical China, seasonal drought during the non-monsoon period contributes to an increase in fire risk [32]. Forest-burned areas are mainly distributed in Northeast China, Southwest China, and South China [37]. The frequency of small fires is high in the southwest region, while the frequency of large fires is low in the northeast region [38]. To explore the future probability of forest fire occurrence across different regions, we divided the country into seven regions based on previous research [34,37–39]. The seven geographic regions include Northeast China (NEC), North China (NC), Central China (CC), East China (EC), South China (SC), Northwest China (NWC), and Southwest China (SWC) (Fig 1).

### 2.2 Data source and pre-processing

#### 2.2.1 Forest fire occurrence probability.

**Fig 2:**
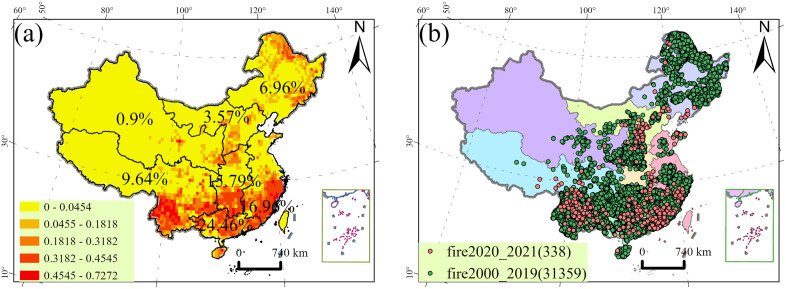
Distribution of forest fire occurrence probability (a) and records by year in China (2000-2021) (b). The percentages in text labels represent the average grid FP for each region. Note: The map is produced based on the standard map with review number GS (2023)2767 downloaded from the website of the Standard Map Service of the State Administration of Surveying of the People’s Republic of China, Mapping and Geoinformation(http://bzdt.ch.mnr.gov.cn/), with no modification to the base map.

Our forest fire record data largely came from the fire archives of China (1999–2019) [40], containing 53561 fire records. We added official fire records from 2020 to 2021 to this archive. We combined the above-mentioned forest fire records to form a forest fire record data set (1999–2021). We did not use those records in 1999 to ensure consistency with the period of the national population distribution data (2000–2021). In addition, we removed those records that have no latitude and longitude. Finally, a total of 31697 records remained for further analysis (Fig 2b). We divided the forest fire records (2000–2021) into 2 subsets, 2000–2019 and 2020–2021. The former (2000–2019), with a total of 31359 fire records, was used to construct prediction models for forest fire occurrence probability, and the latter (2020–2021), with a total of 338 fire records, was employed as an independent test set [41]. We estimated the forest fire occurrence probability (FP) for each grid (0.5° × 0.5°) by dividing the years in which forest fires had occurred at least once in each grid by the total number of years in the fire dataset. The probability of forest fires in China (2000–2021) is shown in Fig 2a.

#### 2.2.2 Natural and anthropogenic variables.

Multiple environmental variables influence forest fire occurrence probability. In addition to the drought characteristics, this paper divides the variables into natural and anthropogenic variables [42]. We collected both natural and anthropogenic variables for each grid (0.5° × 0.5°). Natural variables mainly include river density, slope, elevation, tree cover, and surface roughness. Topographical factors (slope and DEM) can significantly influence wildfire occurrence by altering fuel distribution and flame propagation pathways [36]. Vegetation cover influences the likelihood of forest fire occurrence through fuel abundance [36]. River factors influence forest fire occurrence by affecting the intensity of human activity, with greater human activity occurring closer to rivers [42]. The average slope and elevation for each grid were extracted using ArcGIS Pro based on national 1 km resolution DEM data from the Resource and Environmental Science Data Platform [43]. The tree cover data (2000–2021) from China Annual Tree Cover Dataset (CATCD) [44], we calculated the average tree cover(2000–2019,2020–2021) for each grid. The surface roughness was extracted for each grid by the local elevation difference algorithm [45] with the following formula(1). The river density is obtained according to equation (2). We calculate the roughness by determining the difference between the maximum and minimum elevation points within each grid.


$$R=Z_{\max}-Z_{\min}$$ (1)

Where R is the surface roughness, $Z_{max}$ is the maximum elevation value, and $Z_{min\;}$ is the minimum elevation value in the grid cell.

Anthropogenic variables are characterized by population density and road density. The impact of anthropogenic factors on fire occurrence can be twofold: on the one hand, high population densities increase the likelihood of human-induced fires; on the other hand, they can also help reduce fire occurrences by decreasing fuel availability and altering vegetation patterns [46]. Anthropogenic variables exert varying influences on forest fire occurrence across different regions, exhibiting spatial non-stationarity. In some areas, anthropogenic variables predominantly promote forest fire occurrence, while in others, they primarily mitigate it. Employing geographically weighted logistic regression models enables consideration of the spatial non-stationarity of influencing factors on forest fire occurrence. Based on the national population distribution data (2000–2021) [47–68], the population density (persons/km^2^) (2000–2019,2020–2021) were obtained by dividing the sum of the population distribution within the grid by the area of the grid; Based on the road and river distribution data (scale 1:1,000,000) [69], the road density and river density (2000–2019,2020–2021) were obtained by dividing the length of the roads and rivers within each grid by the area of the grid with the following formula (Equation 2).


δ=L/F (2)

Where: δ is the road or river network density in km/km^2^; L is the road or river network length in km; F is the area of the grid in km^2^.

#### 2.2.3 The standard precipitation evapotranspiration index (SPEI).

The Standardized Precipitation Evapotranspiration Index (SPEI) was used to characterize droughts. SPEI has multiple time scales and can identify droughts at different time scales [70], as well as perform better in assessing the effects of drought on vegetation growth [71]. Current climate SPEI data (2000–2021) comes from the global SPEI database SPEI base v.2.9 [72] with a 0.5-degree spatial resolution, which is in format and needs to be converted to raster format first. Phase 6 of the Coupled Model Intercomparison Project (CMIP6) improved the assessment and prediction of various aspects of climate, such as precipitation, and the mean and extreme temperatures [73]. The Scenario Model Intercomparison Project (Scenario MIP) is a new climate change scenario proposed by the sixth phase of the Coupled Model Intercomparison Project [74]. Future climate SPEI data were calculated from CMIP6 future meteorological data. Meteorological data for future climate conditions were obtained from the National Tibetan Plateau Data Center’s HiCPC: High-resolution CMIP6 downscaled daily climate projections over China 1979–2100 [75] with a spatial resolution of 0.1° × 0.1° and a temporal resolution of one month. Since the HiCPC dataset has already undergone bias correction using the China Meteorological Forcing Dataset (CMFD) as a reference [75], we did not perform bias correction before using this dataset. CMIP6 combines the Shared Socioeconomic Pathway (SSP) and the Representative Concentration Pathway (RCP) to provide realistic projections of future climate change [76]. For example, the SSP245 scenario (i.e., medium emission) is a combination of the SSP2 and RCP4.5 scenarios; SSP585 (high emission) is a combination of SSP5 and RCP8.5 [77]. Climate change is one of the important issues today, and it will continue to be a serious problem as it will increase its impact on the frequency, duration, and intensity of extreme events [78]. Therefore, to assess all possible future scenarios, meteorological data (monthly average temperature, precipitation) for four scenarios, SSP126, SSP245, SSP370, and SSP585, were selected from optimistic to pessimistic (2022–2099). These scenarios represent a sustainability policy scenario with low GHG emission levels, a middle-of-the-road scenario with medium GHG emission levels and slow sustainability policies, a scenario with high GHG emission levels and more regional competition, and a scenario with very high levels of GHG emission and fossil fuel growth policies, respectively [78]. The future period is divided into near-term (2022–2040), medium-term (2041–2060), and long-term (2080–2099). Global climate models (GCMs) play an important role in the study of hydro-climatic extremes and their impacts [79], and in this paper, 15 GCMs were selected (Table 1) and the meteorological data (monthly mean temperature and monthly precipitation) were resampled into a 0.5° × 0.5° grid by bilinear interpolation to match the resolution of the current SPEI data(2000–2021). Given that 15 global climate models (GCMs) provided data for two variables (pr, tas), four future scenarios (SSP126, SSP245, SSP370, SSP585), and the future period (2022–2099), we selected these 15 GCMs. Finally, the multimodal mean ensemble (MME) of the 15 GCMs was adopted as the climate conditions for the future period to reduce the uncertainty of different GCMs [80].

**Table 1:** The detailed information of the CMIP6 climate models used in this study.

Model	Institution	Country
ACCESS-CM2	Commonwealth Scientific and Industrial Research Organization	Australia
ACCESS-ESM1–5	Commonwealth Scientific and Industrial Research Organization	Australia
AWI-CM-1–1-MR	Alfred Wegener Institute	Germany
CESM2	National Center for Atmospheric Research	America
CNRM-CM6–1	Centre National de Recherches Meteorologiques	France
CNRM-ESM2–1	Centre National de Recherches Meteorologiques	France
EC-Earth3-Veg	EC-Earth Consortium	Europe
INM-CM4–8	Institute for Numerical Mathematics	Russia
INM-CM5–0	Institute for Numerical Mathematics	Russia
KACE-1–0-G	National Institute of Meteorological Sciences/Korea Meteorological Administration	Korea
MPI-ESM1–2-HR	Max Planck Institute for Meteorology	Germany
MRI-ESM2–0	Meteorological Research Institute	Japan
Nor ESM2-LM	Norwegian Climate Centre	Norway
Nor ESM2-MM	Norwegian Climate Centre	Norway
TaiESM1	Research Center for Environmental Changes, ACADEMIA SINICA	Taiwan, China

In this paper, monthly mean temperature and monthly precipitation data within each grid were extracted by ArcGIS Pro. SPEI was calculated for each month of every year during 2022–2099 using the R package SPEI for 1-, 3-, 6-, and 12-month time scales. PET is difficult to monitor instrumentally, and is usually calculated using empirical equations, the generally accepted Penman-Monteith and Thornthwaite methods [70]. In this paper, the Thornthwaite method was used to calculate the PET, which has the advantage of requiring only data on the monthly mean temperature [81]. Although the PM equation provides a more comprehensive representation of physical processes [82], the integration of results from different indicators demonstrates that actual changes are significant enough to override biases among these indices [82,83]. The main steps for calculating the SPEI index are described in the literature [84].

#### 2.2.4 Drought characterization.

The paper defines a drought event as a period of at least three consecutive months with SPEI values below −0.5, by China’s meteorological drought classification criteria [85] (Table 2). Thresholds = −0.5 and = −1 represent light and mid drought, respectively. We extracted drought characterization corresponding to light and mid drought to establish forest fire occurrence probability prediction models.

**Table 2:** China’s meteorological drought classification standard.

SPEI	Degree of drought
(−0.5, +∞)	No drought
(−1.0, −0.5]	Light drought
(−1.5, −1.0]	Mid-drought
(−2.0, −1.5]	Heavy drought
(- ∞ , −2.0]	Extraordinary drought

The MATLAB programming language was used to implement the run theory algorithm and to batch calculate the drought characteristics for each grid cell for each year during 2000–2021 and 2022–2099. According to the run theory [86], the drought characteristics (drought duration, drought severity, drought intensity) of each drought event can be calculated according to the following equations [87,88]:


DD=DTT−DIT (3)

$$DS=\sum_{i=1}^{DD}SPEI_{i}$$ (4)

$$DI=\frac{DS}{DD}$$ (5)

where DTT and DIT are the drought end and start times, respectively, ${SPEI}_{i}$ is the standardized precipitation evapotranspiration index (SPEI) value for month i, DD is the drought duration, DS is the drought severity, and DI is the drought intensity.

We divided the period into five periods: historical period (2000–2019), model test period (2020–2021), near-term (2022–2040), medium-term (2041–2060), and long-term (2080–2099) period. DN denotes the drought event number; we also counted the total number of drought events (n) in each grid cell for each period. Then, we calculated the average annual number of drought events per period (MDN), the average duration of each drought event per period (MDD), the average severity of each drought event per period (MDS), and the average intensity of each drought event per period (MDI) (Eqs. 6, 7, 8, 9). MDN and MDD are positive indicators; higher values indicate greater drought number and duration. MDS and MDI are negative indicators; lower values indicate greater drought severity and intensity.


$$MDN=\frac{\sum_{j=1}^{n}DN_{j}}{y}$$ (6)

$$MDD=\frac{\sum_{j=1}^{n}DD_{j}}{n}$$ (7)

$$MDS=\frac{\sum_{j=1}^{n}DS_{j}}{n}$$ (8)

$$MDI=\frac{\sum_{j=1}^{n}DI_{j}}{n}$$ (9)

where *DN*_*j*_ is the jth drought event, *DD*_*j*_ is the duration of the jth drought event, *DS*_*j*_ is the severity of the jth drought event, *DI*_*j*_ is the intensity of the jth drought event, *y* is the number of years for per period. *MDN* is the average annual number of drought events per period. *MDD* is the average duration of each drought event per period, *MDS* is the average severity of each drought event per period, and *MDI* is the average intensity of each drought event per period. n is the total number of drought events per period.

### 2.3 Data analysis methods

#### 2.3.1 Diagnosis of multicollinearity among explanatory variables.

Multicollinearity refers to a high degree of correlation between the explanatory variables in a model, which can lead to distortion or difficulty in estimating the model accurately [89]. VIF is an important measure of the severity of multicollinearity. When VIF ranges from 0 to 10, there is no multicollinearity; however, when VIF ≥ 10, a high degree of multicollinearity exists between the variables, indicating that some variables may need to be removed from the model [90]. We performed the VIF covariance diagnosis of the 11 independent variables at each time scale using R 4.5.0. The results indicate that multicollinearity (VIF ≥ 10) exists among the drought characteristics (MDD, MDS, MDI).

#### 2.3.2 Principal component analysis.

We performed principal component analysis (PCA) to address multicollinearity among drought characteristics. It not only fully considers the contribution of all potential explanatory variables to the changes in the dependent variable but also effectively addresses multicollinearity among explanatory variables [91]. We performed a principal component analysis on drought characteristics, selecting three principal components with cumulative contributions exceeding 90% [91] for inclusion in the prediction model. Furthermore, we transformed drought characteristics for future periods using the same standardized parameters and load matrix. The principal component contribution rates and loading matrix are shown in Table 5.

#### 2.3.3 Data standardization.

Data standardization is necessary to eliminate the quantitative influence of factors affecting the probability of forest fire occurrence, ensuring that the influencing factors are of the same order of magnitude and thus suitable for comprehensive and comparative evaluation. In this paper, the normalization was performed according to the formulas of [92], making the value of the influencing factors limited to the range of [0,1], and the specific explanations are shown in Table 3:

**Table 3:** Normalized formulas and explanations.

Formula	Explanation	Variables Using This Formula
$x_{i}^{*}=\frac{x_{i}-x_{\min}}{x_{\max}-x_{\min}}$	$x_{i}$and $x_{i}^{*}\;$are the values before and after data normalization, respectively; $x_{max}$ and $x_{min}\;$are the maximum and minimum values of the full sample data, respectively.	Slope, terrain roughness, road density, River density, population density, elevation, tree cover, PC1, PC2, PC3

#### 2.3.4 Construction of models for forest fire occurrence probability based on drought characteristics.

We modeled forest fire occurrence probability (FP) at the grid cell scale. The independent variables were PCAs of drought characteristics, along with natural and anthropogenic variables. The models were built for 1-, 3-, 6-, and 12-month temporal scales, respectively. FP was modeled by global logistic regression (GLR) and geographically weighted logistic regression (GWLR) models using GWR4 software [93].

The global regression model does not consider the effect of spatial location and assumes that the regression coefficients of independent variables are spatially invariant, i.e., spatial non-stationarity [94]. The global regression model is formulated as follows:


$$y_{i}=\alpha +\beta x_{i1}+...+\tau x_{in}+\varepsilon_{i}$$ (10)

where y is the dependent variable, x is the independent variable, α, β...τ, are the parameters to be estimated, ε is the error term, and i is a location in space where observations of y and x are recorded.

Previous researches indicate that spatial heterogeneity is common in forest fire occurrence probability [95,96]. The relationship between forest fire occurrence probability and influencing factors can also exhibit significant spatial variation, i.e., the independent variable affects the dependent variable differently across different regions [97,98], and this variation is referred to as spatial non-stationarity. The geographically weighted regression (GWR) model can model the problem of spatial non-stationarity of variables by estimating the regression coefficients of each spatial unit [99]. The expression of GWR at this point is [100]:


$$y_{i}=\sum_{k=1}^{n}\beta_{k}(u_{i},v_{i})x_{ik}+\beta_{0}(u_{i},v_{i})+\varepsilon_{i}$$ (11)

Where, $y_{i}$ is the explanatory variable; $\beta_{\text{0}}(u_{i},v_{i})$ is the intercept; $x_{ik}$ is the kth explanatory variable for sample i; ($u_{i},v_{i}$) are the spatial coordinates of i; $\beta_{k}(u_{i},v_{i})$ is the coefficient of the kth explanatory variable for sample i; $\varepsilon_{i}$ (i=1, 2, 3,...., k) is the random perturbation term.

The logistic regression model maps the results of linear regression to the (0,1) space by a sigmoid function, and the probability of forest fire occurrence (Y = 1) at location *i* is P. The probability of forest fire occurrence at location *i* is [101]:


$$P(Y=1)=\frac{\text{1}}{1+e^{-y_{i}}}$$ (12)

After the Logit transformation, there is


$$\log it(P)=\ln\frac{P}{1-P}=y_{i}$$ (13)

Where, if $y_{i}$ is a global regression model, the equation is a global logistic regression (GLR) model; if $y_{i}$ is a geographically weighted regression model, the equation is a geographically weighted logistic regression (GWLR) model.

In this paper, we used the Adaptive bi-square kernel function to compute the weight matrix and the optimal bandwidth of the model, which is determined using the Akaike information criterion AIC’s calibration value, *AICc*. The formula of *AICc* is as follows [102]:


AIC=−2ln(L)+2k (14)

AICc=AIC+(2k(k+1)/(m−k−1)) (15)

where m is the number of sample points, ln(L) is the value of the log-likelihood function for the maximum likelihood estimation of the model, and k is the number of parameters in the model. smaller values of *AICc* indicate higher model precision and lower correlation between explanatory variables.

#### 2.3.5 Model prediction accuracy assessment.

We used the second subset of forest fire records (2020–2021) as a model test dataset [41]. The performance of forest fire occurrence probability models was evaluated using the Receiver Operating Characteristic (ROC) curve and the area under the ROC curve (AUC) [103]. A perfect model is obtained when AUC = 1, while the model is non-informative when AUC = 0 [104]. AUC values between 0.5 and 0.7 are assessed as weak accuracy performance, between 0.7 and 0.9 are considered moderate and potentially useful, and those greater than 0.9 are assessed as high accuracy and excellent predictive discrimination [105,106]. This study employs the *AICc* statistic to evaluate model goodness-of-fit, where a smaller *AICc* value indicates superior model performance [107]. The Brier score was used to evaluate the predictive performance of the model. A smaller Brier score indicates a smaller prediction error and better prediction performance [108].

#### 2.3.6 Modelling spatial and temporal patterns of forest fire occurrence probability in future periods.

To project forest fire characteristics, drought characteristics (temperature and precipitation) were set to their respective future values under different climate scenarios within the constructed models, enabling the output of future predictions [30]. The spatial and temporal pattern of forest fire occurrence probability in China was predicted by the GWLR model for the near-term (2022–2040), medium-term (2041–2060), and long-term (2080–2099) periods according to the multimodal mean ensemble (MME) data under scenario SSP126, SSP246, SSP370, and SSP585, respectively. The national land use types from the Resource and Environmental Science Data Platform [109] were used to extract forest land, which acts as a mask to calculate the difference in forest fire occurrence probability in each geographic region compared to the historical period (2000–2019) for each scenario during the three time periods. We counted the average change in probability for grids containing forest land in each geographic region.

## 3 Results

### 3.1 Multicollinearity among explanatory variables

Multicollinearity analysis showed that the drought characteristics (MDD, MDS, MDI) were multicollinear at four temporal (1-,3-,6-, 12-month) scales (Table 4). The highest multicollinearity exists between drought duration (MDD) and drought severity (MDS). Drought intensity exhibits significant collinearity (VIF > 10) at the SPEI-1 timescale with a threshold of −0.5. Other influencing factors besides drought characteristics exhibit low collinearity (VIF < 10).

**Table 4:** VIF of explanatory variables for multicollinearity diagnosis.

Threshold	time scale	MDN	MDD	MDS	MDI	population density	Terrain roughness	road density	River density	slope	elevation	Tree cover
−0.5	SPEI-1	1.95	48.23	71.43	18.80	2.38	4.76	3.38	1.90	6.31	3.92	2.54
−0.5	SPEI-3	1.72	63.48	84.41	8.93	2.41	4.77	3.37	1.93	6.31	4.00	2.52
−0.5	SPEI-6	1.56	93.48	111.49	5.04	2.44	4.86	3.40	1.94	6.37	4.00	2.43
−0.5	SPEI-12	1.68	136.76	142.60	2.38	2.44	4.85	3.52	1.81	6.41	3.50	2.31
−1	SPEI-1	1.85	9.44	12.48	2.54	2.38	4.75	3.46	1.80	6.33	3.44	2.42
−1	SPEI-3	2.14	39.51	43.79	5.50	2.42	4.76	3.34	1.92	6.34	3.82	2.48
−1	SPEI-6	1.78	77.08	83.80	5.40	2.46	4.91	3.44	1.94	6.41	3.75	2.41
−1	SPEI-12	1.40	137.65	145.94	2.55	2.49	4.78	3.57	1.91	6.38	3.71	3.37

### 3.2 Principal component analysis

In PC1, MDD and MDS exhibit high loadings, with MDD predominantly showing positive loadings and MDS predominantly showing negative loadings. However, MDD displays negative loadings and MDS shows positive ones at the drought threshold of −0.5 and the SPEI-12 timescale (Table 5).

**Table 5:** Variance proportion of principal components (%) and Loadings for drought characteristics.

Time scale	Drought Characteristics	Threshold	PC1	PC2	PC3	Threshold	PC1	PC2	PC3
SPEI-1	MDN	−0.5	0.44	−0.03	0.9	−1	0.25	0.96	−0.07
	MDD		0.53	−0.54	−0.28		0.57	−0.1	0.52
	MDS		−0.62	0.03	0.3		−0.59	0.15	−0.23
	MDI		−0.37	−0.84	0.16		−0.51	0.19	0.82
	Proportion of Variance (%)		60.29	22.86	16.67		66.38	22.23	10.17
	Cumulative Proportion (%)		60.29	83.14	99.81		66.38	88.61	98.78
SPEI-3	MDN		0.37	0.63	−0.68		0.43	0.45	−0.78
	MDD		0.58	0.16	0.47		0.63	0.07	0.38
	MDS		−0.62	0.08	−0.26		−0.63	0.21	−0.24
	MDI		−0.37	0.76	0.49		−0.13	0.86	0.44
	Proportion of Variance (%)		61.11	22.20	16.53		57.21	28.80	13.70
	Cumulative Proportion (%)		61.11	83.31	99.83		57.21	86.01	99.70
SPEI-6	MDN		0.18	0.87	0.46		0.4	−0.39	0.83
	MDD		0.61	−0.29	0.31		0.63	−0.11	−0.35
	MDS		−0.64	0.2	−0.13		−0.64	−0.11	0.26
	MDI		−0.43	−0.35	0.82		−0.17	−0.91	−0.34
	Proportion of Variance (%)		57.29	26.08	16.50		56.47	25.78	17.60
	Cumulative Proportion (%)		57.29	83.37	99.87		56.47	82.25	99.85
SPEI-12	MDN		0.41	−0.46	−0.79		0.05	0.84	0.55
	MDD		−0.58	−0.43	−0.03		0.67	0.11	−0.23
	MDS		0.59	0.37	0.1		−0.68	−0.05	0.14
	MDI		0.4	−0.69	0.6		−0.28	0.53	−0.79
	Proportion of Variance (%)		65.34	17.78	16.79		52.33	26.62	20.96
	Cumulative Proportion (%)		65.34	83.12	99.91		52.33	78.95	99.91

In PC2, at the drought threshold of −0.5, MDI exhibits significant negative loadings at the SPEI-1 and SPEI-12 timescales, while showing significant positive loadings at the SPEI-3 timescale. At the SPEI-6 timescale, MDN displays significant positive loadings. At a drought threshold of −1, MDN exhibits significant positive loadings at the SPEI-1 and SPEI-12 timescales. At the SPEI-3 timescale, MDI shows significant positive loadings, while at the SPEI-6 timescale, it exhibits significant negative loadings.

In PC3, at the drought threshold of −0.5, MDN exhibits significant negative loadings at the SPEI-3 and SPEI-12 timescales, while showing significant positive loadings at the SPEI-1 timescale. At the SPEI-6 timescale, MDI displays significant positive loadings. At a drought threshold of −1, MDI exhibits large positive loadings at the SPEI-1 timescale and large negative loadings at the SPEI-12 timescale. MDN shows large negative loadings at the SPEI-3 timescale and a large positive load at the SPEI-6 timescale.

### 3.3 Spatial and temporal changes of future drought characteristics

**Fig 3:**
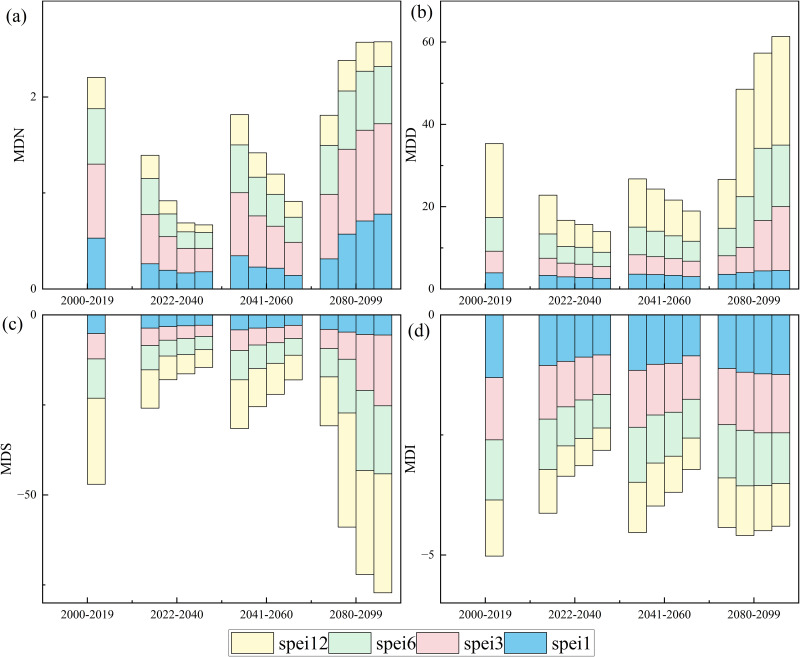
Bar chart showing changes in the average national light drought characteristics (Threshold = −0.5). The bars within each future period are shown from left to right for the SSP126, SSP245, SSP370, and SSP585, respectively.

**Fig 4:**
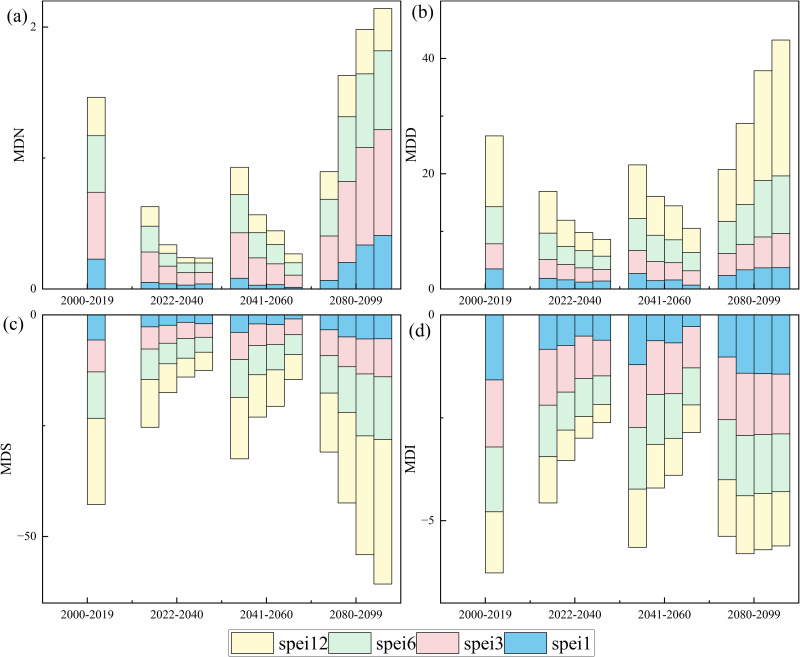
Bar chart showing changes in the average national mid drought characteristics (Threshold = −1). The bars within each future period are shown from left to right for the SSP126, SSP245, SSP370, and SSP585, respectively.

We analyzed changes in national patterns of mild drought (Threshold = −0.5) (Fig 3) and moderate drought (Threshold = −1) (Fig 4). Under the SSP126 scenario, MDN, MDI, MDD, and MDS decreased at all time scales and future periods, indicating a wetting trend in the future. Under the SSP245, SSP370, and SSP585 scenarios, MDN, MDD, MDS, and MDI broadly showed a trend of decreasing in the near- and medium-term, and increasing in the long-term. Under SSP585 conditions, the changes are more pronounced, with more significant moist in the near and medium term and drier conditions in the long term.

### 3.4 Goodness of fit for forest fire occurrence prediction models

Table 6 lists the indicators used to assess the performance of the models in predicting the probability of forest fire occurrences. Based on AUC evaluation at the drought threshold of −0.5, the SPEI-3 and SPEI-6 timescales yielded the best prediction models. At a drought threshold of −1, the SPEI-1 and SPEI-12 timescales produced the best prediction models. Based on *AICc* evaluation at the drought threshold of −0.5, the SPEI-1 and SPEI-6 timescales yielded the best model fitting. At a drought threshold of −1, the SPEI-6 and SPEI-12 timescales produced the best model fitting. Based on Brier Score evaluation at both the drought thresholds of −0.5 and −1, the SPEI-1 and SPEI-12 timescales yielded the best prediction models.

**Table 6:** Comparison of the goodness of fit of forest fire occurrence probability models by test dataset.

	Time scale	Models	AUC	Significance test	*AICc*	Brier Score
0.5	1	GWLR	0.7886	P < 0.001	5467.13	0.062246
0.5	3	GWLR	0.8085	P < 0.001	5496.85	0.063813
0.5	6	GWLR	0.7953	P < 0.001	5470.43	0.066873
0.5	12	GWLR	0.779	P < 0.001	5477.72	0.052782
1	1	GWLR	0.8252	P < 0.001	5512.91	0.057662
1	3	GWLR	0.7757	P < 0.001	5526.74	0.066149
1	6	GWLR	0.7631	P < 0.001	5499.64	0.086366
1	12	GWLR	0.7838	P < 0.001	5507.74	0.05143

### 3.5 Relative importance of independent variables

Table 7 shows that the main factors influencing the probability of forest fire occurrence in Central China are Tree cover, Terrain roughness, Road density, and Slope. At the SPEI-6 (threshold = −0.5, threshold = − 1) and SPEI-12 (threshold = − 1) timescale, PC1 exhibits a significant positive correlation in this region, while MDS shows substantial negative loadings on PC1. The PC1 indicates that the greater the drought severity, the higher the probability of forest fire occurrence. At the SPEI-12 (threshold = −0.5) timescale, PC1 exhibits a significant negative correlation in this region, while MDS shows substantial positive loadings on PC1. The PC1 indicates that the greater the drought severity, the higher the probability of forest fire occurrence. At the SPEI-1 (threshold = −0.5) timescale, PC3 exhibits a significant negative correlation in this region, while MDN shows substantial positive loadings on PC3. The PC3 indicates that the higher the drought number, the lower the probability of forest fire occurrence. At the SPEI-3 (threshold = −0.5) timescale, PC3 exhibits a significant positive correlation in this region, while MDN shows substantial negative loadings on PC3. The PC3 indicates that the higher the drought number, the lower the probability of forest fire occurrence. At the SPEI-1 (threshold = −1) timescale, PC3 exhibits a significant positive correlation in this region, while MDI shows substantial positive loadings on PC3. The PC3 indicates that the greater the drought intensity, the lower the probability of forest fire occurrence. At the SPEI-3 (threshold = −1) timescale, PC2 exhibits a significant negative correlation in this region, while MDI shows substantial positive loadings on PC2. The PC2 indicates that the greater the drought intensity, the higher the probability of forest fire occurrence.

**Table 7:** Mean coefficients of the independent variables in various regions at different time scales.

Region	Time scales	Threshold	Eevation	Tree cover	Population density	Terrain roughness	Road density	River density	Slope	PC1	PC2	PC3
CC	SPEI1	−0.5	−0.14	3.54	−1.71	3.94	3.01	0.97	−4.76	−0.21	1.64	−3.67
CC	SPEI3	−0.5	−0.25	3.12	−2.69	3.89	3.78	1.04	−4.21	4.59	−10.33	43.01
CC	SPEI6	−0.5	−0.23	3.02	−1.86	3.26	3.56	1.31	−3.08	25.53	−20.68	2.43
CC	SPEI12	−0.5	−0.88	3.2	−1.22	3.5	3.03	1.35	−3.41	−6.74	0.58	−3.43
CC	SPEI1	−1	0.33	3.49	−1.99	3.48	3.31	1.18	−4.22	0.67	−0.57	1.79
CC	SPEI3	−1	−0.01	3.48	−1.97	3.62	3.5	1.04	−3.97	0.66	−5.87	−0.12
CC	SPEI6	−1	−0.91	3.05	−1.21	3.73	2.84	1.3	−3.83	30.01	−2.93	−5.26
CC	SPEI12	−1	−0.84	3.21	−1.3	3.44	2.97	1.5	−3.37	14.95	0.71	2.61
EC	SPEI1	−0.5	−1.12	3.67	−2.25	3.24	3.74	0.98	−3.57	−0.77	1.65	−2.21
EC	SPEI3	−0.5	−0.75	3.08	−3.07	3.71	4.15	1.17	−3.72	4.2	−11.48	39.38
EC	SPEI6	−0.5	−0.95	3.04	−2.59	2.93	4.05	1.27	−2.62	26.18	−16.98	−0.27
EC	SPEI12	−0.5	−2.44	3.06	−1.66	3.35	3.32	1.31	−2.54	−5.81	1.43	−5.32
EC	SPEI1	−1	−0.27	3.48	−2.17	3.17	3.49	1.15	−3.7	0.04	−1.83	0.96
EC	SPEI3	−1	−0.55	3.42	−2.09	3.42	3.63	1.17	−3.29	−0.21	−6.61	−1.62
EC	SPEI6	−1	−2.26	3.05	−2.24	3.06	3.71	1.14	−2.5	28.13	−2.62	−3.82
EC	SPEI12	−1	−2.05	3.19	−1.59	3.28	3.23	1.26	−2.78	8.04	−0.01	6.35
NC	SPEI1	−0.5	0.52	2.37	−4.88	4.73	6.83	−0.67	−3.32	−4.61	−1.23	1.07
NC	SPEI3	−0.5	0.51	2.47	−4.51	4.7	6.49	−0.15	−3.26	−13.74	3.64	14.78
NC	SPEI6	−0.5	0.21	2.46	−4.71	4.35	6.33	−0.09	−3.09	−10.09	−2.48	5.44
NC	SPEI12	−0.5	−0.18	2.3	−4.5	4.04	6.05	−0.49	−3.02	9.29	4.09	−2.02
NC	SPEI1	−1	0.04	2.48	−4.65	4.11	6.45	−0.32	−2.81	2.06	−3.48	−1.39
NC	SPEI3	−1	0.12	2.43	−4.71	4.61	6.37	−0.28	−3.29	−4.23	−1	3.15
NC	SPEI6	−1	0.24	2.08	−5.48	4.6	6.89	−0.81	−3.06	−11.34	−4.19	−10.81
NC	SPEI12	−1	−0.05	2.49	−4.66	4.29	6.15	−0.33	−3.2	−12.47	−1.84	4.47
NEC	SPEI1	−0.5	−0.63	1.73	−4.47	3.55	2.86	0.24	−2.72	−5.91	−0.61	−4.5
NEC	SPEI3	−0.5	−1.26	1.79	−3.47	4.66	3.88	−0.32	−2.67	−51.43	−12.72	33.63
NEC	SPEI6	−0.5	−1.1	2.16	−3.7	4.75	3.96	−0.27	−3.41	−19.96	−3.68	−18.99
NEC	SPEI12	−0.5	−1.66	2.37	−4.31	4.32	3.51	0.3	−3.94	6.01	7.97	0.05
NEC	SPEI1	−1	−1.83	2.04	−5.29	3.33	3.17	0.41	−3	−1.33	−3.47	−2.2
NEC	SPEI3	−1	−0.95	2.09	−4.88	3.77	3.58	0.31	−3.29	−6.64	−2.38	4.88
NEC	SPEI6	−1	−0.59	1.94	−5.93	3.34	3.12	0.36	−3.5	−19.19	1.14	−1.33
NEC	SPEI12	−1	−1.65	2.32	−4.97	3.57	3.46	0.48	−3.31	−12.66	−2.03	4.1
NWC	SPEI1	−0.5	2.88	7	−9.09	0.27	14.75	−2.75	−1.71	−18.16	6.1	0.25
NWC	SPEI3	−0.5	3.24	5.01	−9.37	−0.43	14.43	−2.71	0.38	0.27	7.06	45.61
NWC	SPEI6	−0.5	3.09	4.95	−9.14	−0.57	14.05	−2.46	0.73	−0.04	−3.61	11.62
NWC	SPEI12	−0.5	3.03	4.72	−9.68	−1.07	14.1	−2.98	0.8	5.28	−2.59	−2.44
NWC	SPEI1	−1	3.16	5.35	−7.62	−0.44	14.06	−2.41	0.41	6.96	−0.3	−1.71
NWC	SPEI3	−1	2.11	4.7	−8.43	−0.21	14.3	−3.7	0.39	−1.71	−7.08	8.36
NWC	SPEI6	−1	3.01	6.48	−7.93	0.58	14.8	−2.44	−1.25	−17.88	6.19	−6.52
NWC	SPEI12	−1	2.93	5.06	−8.74	−0.3	14.23	−2.71	0.51	2.67	0.08	−1.34
SC	SPEI1	−0.5	0.33	3.04	−1.48	2.03	2.52	1.05	−3.07	4	−0.53	−2.49
SC	SPEI3	−0.5	−0.09	2.71	−2.84	1.89	3.64	0.86	−2.63	24.09	−8.24	43.55
SC	SPEI6	−0.5	−0.09	2.65	−1.43	1.08	2.93	1.08	−1.59	35.18	−18.29	7.26
SC	SPEI12	−0.5	−0.47	2.74	−1.57	1.89	2.96	1.12	−2.16	−7.99	0.29	−2.53
SC	SPEI1	−1	0.43	3.11	−2.43	1.76	3.42	1.09	−2.74	1.25	2.71	2.08
SC	SPEI3	−1	0.15	3.11	−2.11	2.08	3.31	0.97	−2.75	3.92	−2.94	1.42
SC	SPEI6	−1	−0.31	2.45	−1.21	1.29	2.14	1.27	−2.07	31.66	−4.05	−1.39
SC	SPEI12	−1	−0.66	2.64	−1.6	1.68	2.8	1.5	−1.76	20.44	2.46	1.38
SWC	SPEI1	−0.5	0.51	4.1	−0.65	−1.63	9.01	−4.77	0.8	6.39	−3.14	−6.67
SWC	SPEI3	−0.5	0.92	4.16	−8.06	−1.08	9.71	−3.48	0.34	43.64	5.11	59.1
SWC	SPEI6	−0.5	0.95	4.25	−6.25	−1.4	8.98	−3.18	0.96	30.13	−17.75	19.67
SWC	SPEI12	−0.5	0.6	4.3	−7.67	−0.81	9.87	−4.06	0.62	−6.22	−1.84	1.25
SWC	SPEI1	−1	0.79	4.18	−7.3	−1.08	9.63	−3.28	0.52	5.5	3.86	1.93
SWC	SPEI3	−1	0.56	3.98	−8.57	−0.8	9.79	−4.03	0.29	7.68	−0.04	5.91
SWC	SPEI6	−1	1.04	4.22	2.6	−2.16	8.77	−4.49	1.1	28.36	−4.51	−1.75
SWC	SPEI12	−1	0.7	4.32	−7.79	−0.94	9.98	−3.47	0.66	16.39	2.87	−3.45

The main factors influencing the probability of forest fire occurrence in East China were Tree cover, Terrain roughness, Road density, Population density, and Slope. At the SPEI-6 (threshold = −0.5, threshold = − 1) and SPEI-12 (threshold = − 1) timescale, PC1 exhibits a significant positive correlation in this region, while MDS shows substantial negative loadings on PC1. The PC1 indicates that the greater the drought severity, the higher the probability of forest fire occurrence. At the SPEI-12 (threshold = −0.5) timescale, PC1 exhibits a significant negative correlation in this region, while MDS shows substantial positive loadings on PC1. The PC1 indicates that the greater the drought severity, the higher the probability of forest fire occurrence. At the SPEI-1 (threshold = −0.5) timescale, PC3 exhibits a significant negative correlation in this region, while MDN shows substantial positive loadings on PC3. The PC3 indicates that the higher the drought number, the lower the probability of forest fire occurrence. At the SPEI-3 (threshold = −0.5) timescale, PC3 exhibits a significant positive correlation in this region, while MDN shows substantial negative loadings on PC3. The PC3 indicates that the higher the drought number, the lower the probability of forest fire occurrence. At the SPEI-1 (threshold = −1) timescale, PC2 exhibits a significant negative correlation in this region, while MDN shows substantial positive loadings on PC2. The PC2 indicates that the higher the drought number, the lower the probability of forest fire occurrence. At the SPEI-3 (threshold = −1) timescale, PC2 exhibits a significant negative correlation in this region, while MDI shows substantial positive loadings on PC2. The PC2 indicates that the greater the drought intensity, the higher the probability of forest fire occurrence.

The main factors affecting the probability of forest fire occurrence in North China are Tree cover, Population density, Terrain roughness, Road density, and Slope. At the SPEI-1(threshold = −0.5), SPEI-3(threshold = −1), SPEI-6 (threshold = −0.5, threshold = − 1), and SPEI-12 (threshold = − 1) timescales, PC1 exhibits a significant negative correlation in this region, while MDS shows substantial negative loadings on PC1. The PC1 indicates that the greater the drought severity, the lower the probability of forest fire occurrence. At the SPEI-12 (threshold = −0.5) timescale, PC1 exhibits a significant positive correlation in this region, while MDS shows substantial positive loadings on PC1. The PC1 indicates that the greater the drought severity, the lower the probability of forest fire occurrence. At the SPEI-3 (threshold = −0.5) timescale, PC3 exhibits a significant positive correlation in this region, while MDN shows substantial negative loadings on PC3. The PC3 indicates that the higher the drought number, the lower the probability of forest fire occurrence. At the SPEI-1 (threshold = −1) timescale, PC2 exhibits a significant negative correlation in this region, while MDN shows substantial positive loadings on PC2. The PC2 indicates that the higher the drought number, the lower the probability of forest fire occurrence.

Population density, Terrain roughness, Road density, and Slope were the main factors affecting the probability of forest fire in Northeast China. At the SPEI-1(threshold = −0.5), SPEI-3(threshold = −0.5, threshold = − 1), SPEI-6 (threshold = −0.5, threshold = − 1), and SPEI-12 (threshold = − 1) timescales, PC1 exhibits a significant negative correlation in this region, while MDS shows substantial negative loadings on PC1. The PC1 indicates that the greater the drought severity, the lower the probability of forest fire occurrence. At the SPEI-12 (threshold = −0.5) timescale, PC2 exhibits a significant positive correlation in this region, while MDI shows substantial negative loadings on PC2. The PC2 indicates that the greater the drought Intensity, the higher the probability of forest fire occurrence. At the SPEI-1 (threshold = −1) timescale, PC2 exhibits a significant negative correlation in this region, while MDN shows substantial positive loadings on PC2. The PC2 indicates that the higher the drought number, the lower the probability of forest fire occurrence.

Tree cover, population density, road density, and elevation were the main factors influencing forest fire probability in the NWC. At the SPEI-1 (threshold = −1) and SPEI-12 (threshold = −1) timescale, PC1 exhibits a significant positive correlation in this region, while MDS shows substantial negative loadings on PC1. The PC1 indicates that the greater the drought severity, the higher the probability of forest fire occurrence. At the SPEI-12(threshold = −0.5) timescale, PC1 exhibits a significant positive correlation in this region, while MDS shows substantial positive loadings on PC1. The PC1 indicates that the greater the drought severity, the lower the probability of forest fire occurrence. At the SPEI-1 (threshold = −0.5) and SPEI-6 (threshold = −1) timescale, PC1 exhibits a significant negative correlation in this region, while MDS shows substantial negative loadings on PC1. The PC1 indicates that the greater the drought severity, the lower the probability of forest fire occurrence. At the SPEI-3 (threshold = −0.5) timescale, PC3 exhibits a significant positive correlation in this region, while MDN shows substantial negative loadings on PC3. The PC3 indicates that the higher the drought number, the lower the probability of forest fire occurrence. At the SPEI-6 (threshold = −0.5) timescale, PC3 exhibits a significant positive correlation in this region, while MDI shows substantial positive loadings on PC3. The PC3 indicates that the greater the drought intensity, the lower the probability of forest fire occurrence. At the SPEI-3 (threshold = −1) timescale, PC3 exhibits a significant positive correlation in this region, while MDN shows substantial negative loadings on PC3. The PC3 indicates that the higher the drought number, the lower the probability of forest fire occurrence.

The main factors influencing the probability of forest fire occurrence in South China were Tree cover, population density, Road density, and Slope. At the SPEI-1 (threshold = −0.5), SPEI-3 (Threshold = −1), SPEI-6 (Threshold = −0.5, Threshold = − 1), and SPEI-12 (Threshold = − 1) timescales, PC1 exhibits a significant positive correlation in this region, while MDS shows substantial negative loadings on PC1. The PC1 indicates that the greater the drought severity, the higher the probability of forest fire occurrence. At the SPEI-12 (Threshold = −0.5) timescale, PC1 exhibits a significant negative correlation in this region, while MDS shows substantial positive loadings on PC1. The PC1 indicates that the greater the drought severity, the higher the probability of forest fire occurrence. At the SPEI-3 (Threshold = −0.5) timescale, PC3 exhibits a significant positive correlation in this region, while MDN shows substantial negative loadings on PC3. The PC3 indicates that the higher the drought number, the lower the probability of forest fire occurrence. At the SPEI-1 (Threshold = −1) timescale, PC2 exhibits a significant positive correlation in this region, while MDN shows substantial positive loadings on PC2. The PC2 indicates that the higher the drought number, the higher the probability of forest fire occurrence.

The main factors influencing the probability of forest fire occurrence in the Southwest region are Tree cover, Population density, Road density, and River density. At the SPEI-1 (Threshold = −1), SPEI-3 (Threshold = −1), SPEI-6 (Threshold = −0.5, Threshold = − 1), and SPEI-12 (Threshold = − 1) timescales, PC1 exhibits a significant positive correlation in this region, while MDS shows substantial negative loadings on PC1. The PC1 indicates that the greater the drought severity, the higher the probability of forest fire occurrence. At the SPEI-12 (Threshold = −0.5) timescale, PC1 exhibits a significant negative correlation in this region, while MDS shows substantial positive loadings on PC1. The PC1 indicates that the greater the drought severity, the higher the probability of forest fire occurrence. At the SPEI-3 (Threshold = −0.5) timescale, PC3 exhibits a significant positive correlation in this region, while MDN shows substantial negative loadings on PC3. The PC3 indicates that the higher the drought number, the lower the probability of forest fire occurrence. At the SPEI-1 (Threshold = −0.5) timescale, PC3 exhibits a significant negative correlation in this region, while MDN shows substantial positive loadings on PC3. The PC3 indicates that the higher the drought number, the lower the probability of forest fire occurrence.

By examining the sign of the most influential principal component regression coefficient and the sign of the most influential drought characteristics loading, we determined that drought characteristics exhibit a predominantly promoting effect on forest fire occurrence probability in southern China and a predominantly inhibiting effect on forest fire occurrence probability in northern China.

### 3.6 Spatial and temporal patterns of future forest fire occurrence probability

**Fig 5:**
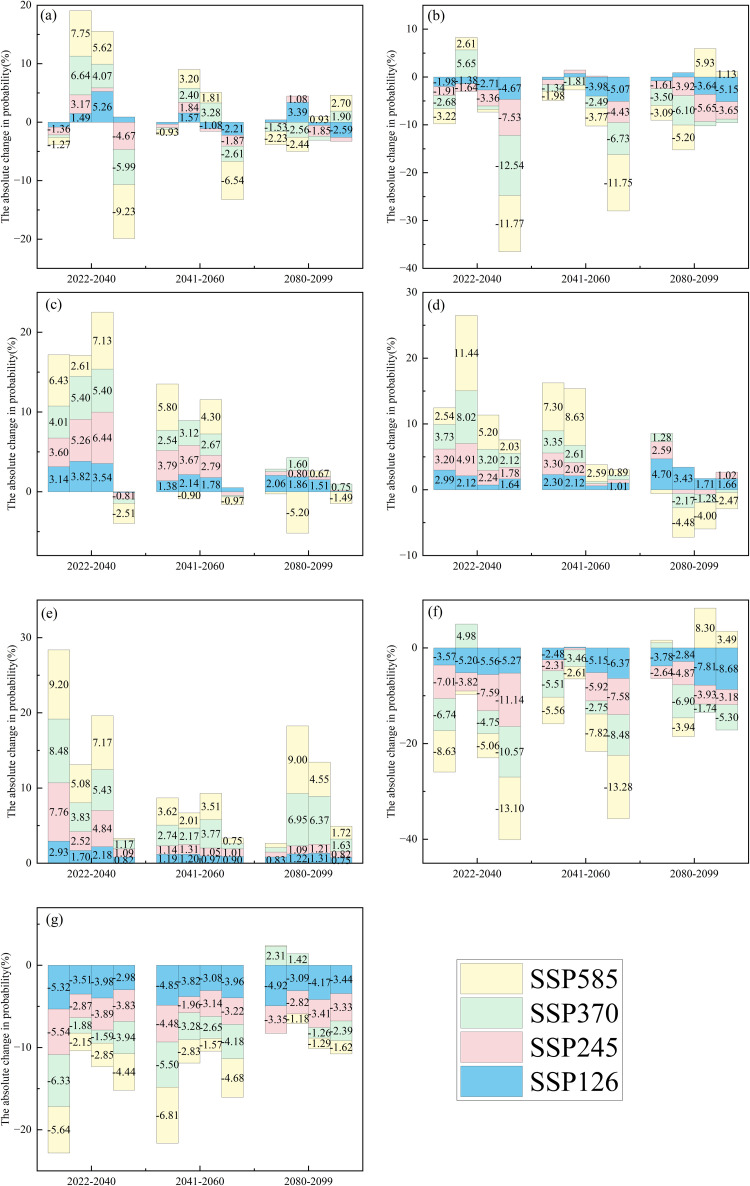
Bar chart showing changes (future period – historical period (2000−2019)) in forest fire occurrence probability for CC(a), EC(b), NC(c), NEC(d), NWC(e), SC(f), and SWC(g). Each future period from left to right is SPEI-1, SPEI-3, SPEI-6, and SPEI-12, respectively. The drought threshold is −0.5. Labels represent absolute probability changes. If the bar height is less than 2%, hide the label.

**Fig 6:**
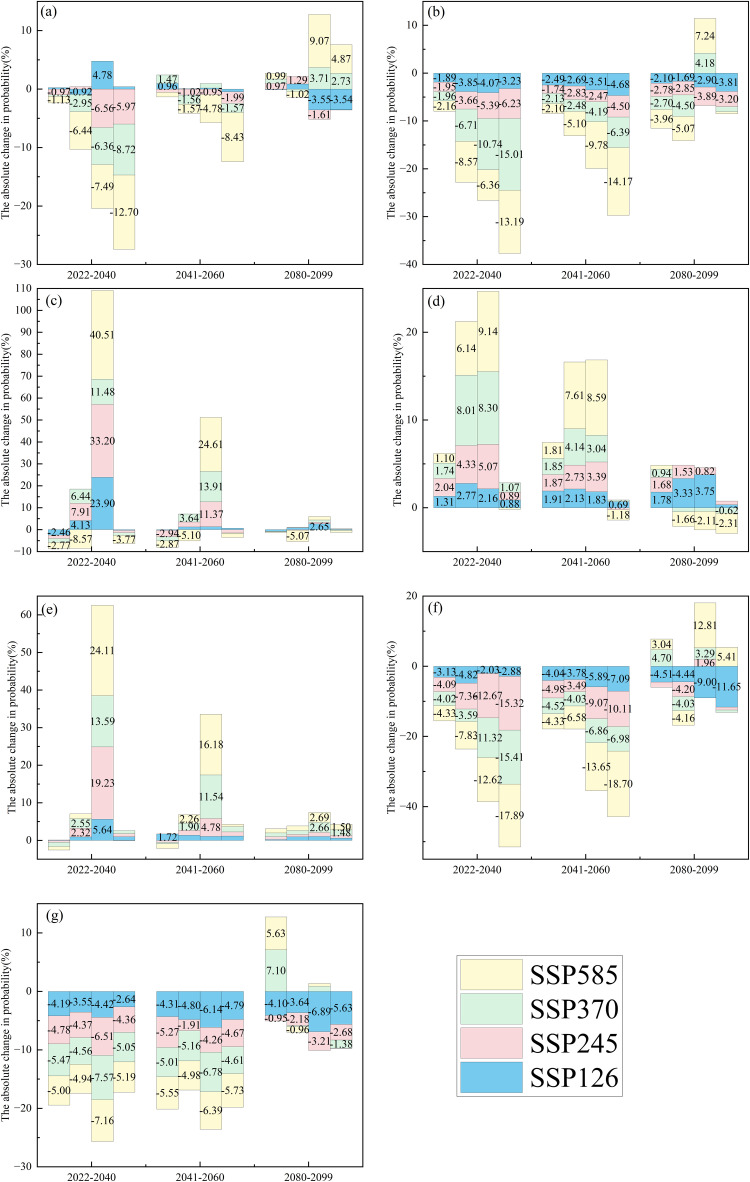
Bar chart showing changes (future period – historical period (2000−2019)) in forest fire occurrence probability for CC(a), EC(b), NC(c), NEC(d), NWC(e), SC(f), and SWC(g). Each future period from left to right is SPEI-1, SPEI-3, SPEI-6, and SPEI-12, respectively. The drought threshold is −1. Labels represent absolute probability changes. If the bar height is less than 2%, hide the label.

Compared to the reference period, regional differences were observed in the changes to forest fire occurrence probability under various scenarios for different future periods and time scales (Figs 5−6). The upward trend in future forest fire occurrence probability is primarily observed in the NC, NEC, and NWC regions, while the downward trend is mainly in the EC, SC, and SWC regions. In the Northeast region (NEC), most timescales and future scenarios under both drought thresholds show an upward trend. In contrast, the Northwest (NWC) and North China (NC) exhibit less pronounced upward trends for SPEI-1 and SPEI-12 timescales at a drought threshold of −1.

At the drought characteristic threshold of −0.5, the probability of forest fire occurrence in Central China (CC) (Fig 5a) has predominantly increased across both the near-term and medium-term at the SPEI-3 and SPEI-6 timescales. The near-term increase ranges from approximately 1.49% to 7.75%, while the medium-term increase ranges from approximately 1.57% to 3.28%. At the SPEI-1 and SPEI-12 timescales, the probability of forest fire occurrence predominantly decreased, with a near-term decrease ranging from approximately 1.27% to 9.23% and a medium-term decrease of about 0.93% to 6.54%. At the drought characteristic threshold of −1, the probability of forest fire occurrence in Central China (CC) (Fig 6a) has predominantly decreased across both the near-term and medium-term. The near-term decrease ranges from approximately 0.97% to 12.70%, while the medium-term decrease ranges from approximately 0.95% to 8.43%.

At the drought characteristic threshold of −0.5, the probability of forest fire occurrence in East China (EC) (Fig 5b) has predominantly decreased across both the near-term, medium-term, and long-term. The near-term decrease ranges from approximately 1.38% to 11.77%, and the medium-term decrease ranges from approximately 1.34% to 11.75%, while the long-term decrease ranges from approximately 1.61% to 6.10%. At the drought characteristic threshold of −1, the probability of forest fire occurrence in East China (EC) (Fig 6b) has predominantly decreased across both the near-term, medium-term, and long-term. The near-term decrease ranges from approximately 1.89% to 13.19%, and the medium-term decrease ranges from approximately 1.74% to 14.17%, while the long-term decrease ranges from approximately 1.69% to 5.07%.

At the drought characteristic threshold of −0.5, the probability of forest fire occurrence in NC (Fig 5c) has predominantly increased across both the near-term and medium-term at the SPEI-1, SPEI-3, and SPEI-6 timescales. The near-term increase ranges from approximately 2.61% to 7.13%, while the medium-term increase ranges from approximately 1.38% to 5.80%. At the drought characteristic threshold of −1, the probability of forest fire occurrence in NC (Fig 6c) has predominantly increased across both the near-term and medium-term at the SPEI-3 and SPEI-6 timescales. The near-term increase ranges from approximately 4.13% to 40.51%, while the medium-term increase ranges from approximately 3.64% to 24.61%.

At the drought characteristic threshold of −0.5, the probability of forest fire occurrence in NEC (Fig 5d) has predominantly increased across both the near-term and medium-term. The near-term increase ranges from approximately 1.64% to 11.44%, while the medium-term increase ranges from approximately 0.89% to 8.63%. At the drought characteristic threshold of −1, the probability of forest fire occurrence in NEC (Fig 6d) has predominantly increased across both the near-term and medium-term. The near-term increase ranges from approximately 0.88% to 9.14%, while the medium-term increase ranges from approximately 0.69% to 8.59%.

At the drought characteristic threshold of −0.5, the probability of forest fire occurrence in NWC (Fig 5e) has predominantly increased across both the near-term, medium-term, and long-term. The near-term increase ranges from approximately 0.82% to 9.20%, the medium-term increase ranges from approximately 0.75% to 3.77%, while the long-term increase ranges from approximately 0.75% to 9%. At the drought characteristic threshold of −1, the probability of forest fire occurrence in NWC (Fig 6e) has predominantly increased across both the near-term, medium-term, and long-term. The near-term increase ranges from approximately 2.32% to 24.11% except for SPEI-1, the medium-term increase ranges from approximately 1.72% to 16.18%, while the long-term increase ranges from approximately 1.48% to 2.69%.

At the drought characteristic threshold of −0.5, the probability of forest fire occurrence in SC (Fig 5f) has predominantly increased across both the near-term, medium-term, and long-term. The near-term decrease ranges from approximately 3.57% to 13.10%, the medium-term decrease ranges from approximately 2.31% to 13.28%, while the long-term decrease ranges from approximately 1.74% to 8.68%. At the drought characteristic threshold of −1, the probability of forest fire occurrence in SC (Fig 6f) has predominantly increased across both the near-term, medium-term. The near-term decrease ranges from approximately 2.03% to 17.89%, while the medium-term decrease ranges from approximately 3.49% to 18.70%.

At the drought characteristic threshold of −0.5, the probability of forest fire occurrence in SWC (Fig 5g) has predominantly increased across both the near-term, medium-term, and long-term. The near-term decrease ranges from approximately 1.88% to 6.33%, the medium-term decrease ranges from approximately 1.57% to 6.81%, while the long-term decrease ranges from approximately 1.18% to 4.92%. At the drought characteristic threshold of −1, the probability of forest fire occurrence in SWC (Fig 6g) has predominantly increased across both the near-term, medium-term. The near-term decrease ranges from approximately 2.64% to 7.57%, while the medium-term decrease ranges from approximately 1.91% to 6.78%.

## 4 Discussion

### 4.1 Spatial and temporal patterns of future drought characteristics

Droughts are the complex interactions among precipitation, temperature, and evapotranspiration [110–115]. Changes in these meteorological variables in climate change scenarios will affect future drought conditions. We quantified MDI, MDS, MDD, and MDN for various emission scenarios at different time scales using SPEI (Figs 3 and 4). We found that under the SSP126 scenario at all four temporal scales, the majority of regions of China generally show a wetting trend as indicated by the decrease of MDI, MDS, MDD, and MDN. This is evidenced by climate change predictions with low emissions always corresponding to wetter conditions [116]. This result is consistent with previous studies [117].

We also found that under the SSP245, SSP370, and SSP585 scenarios, the majority of regions of China broadly show a trend of decreasing drought in the near- and medium-term. Our findings are consistent with previous studies, which showed less frequent droughts in the future across China [21,118,119] due to a significant increase in precipitation [120,121]. More frequent precipitation extremes may also lead to an increase in drought conditions [122,123]. However, the aridity increased in the long term, and the drought severity increases with the emission level. This result is consistent with previous studies [2], which show that higher emission pathways lead to more severe droughts. But SSP585 is considered a highly unlikely pathway and is not recommended for extreme climate impact studies [124].

### 4.2 Model evaluation

This study effectively accounts for the influence of all drought characteristics on forest fire occurrence probability by extracting their principal components and incorporating them into the prediction model. The influence of drought characteristics on forest fire occurrence probability varies across different regions. Moreover, within the same region, different drought characteristics exert differing impacts on forest fire occurrence probability. By establishing the geographically weighted logistic regression prediction model for forest fire occurrence probability based on drought characteristics, this study incorporates the spatial non-stationarity of drought characteristics’ influence on forest fire occurrence probability. This approach overcomes the limitations of linear forest fire occurrence prediction models that solely rely on drought indices.

We modeled forest fire occurrence probability by geographically weighted logistic models that capture local variation in the effects of explanatory variables. We found that the AUC of the GWLR forest fire occurrence probability prediction model was greater than 0.75 at all scales, which indicated that our constructed model predicted better. Our results are consistent with previous studies that have demonstrated the potential of GWLR in modeling large-scale fire occurrence [46,125]. Based on the AUC, AICc, and Brier Score, the SPEI-1 and SPEI-12 timescales demonstrate better model fit and predictive performance. According to the Brier Score, the model’s predictive accuracy decreases as the SPEI time scale increases from 1 to 6 months. The reason for this is discussed by Yin et al. (2024), who noted that the drought–wildfire relationship tended to strengthen significantly when drought conditions lasted 1–3 months but disappeared after more than 3 months in subtropical China [32]. However, time scales of 6–12 months in the SPEI work well in describing drought, and an increase in the length of the filters used in SPEI calculations could reduce the noise more efficiently [126]. Other related studies have also shown that drought indicators derived from the SPEI at longer time scales are significantly more applicable than those derived at shorter time scales [127].

### 4.3 Spatial and temporal patterns of future forest fire occurrence probability

Our results showed regional variations in future forest fire occurrences, increasing in northern China (NC, NEC, and NWC) while decreasing in southern China, especially in SC and SWC. We determined that drought characteristics exhibit a predominantly promoting effect on forest fire occurrence probability in southern China and a predominantly inhibiting effect on forest fire occurrence probability in northern China. This may be related to factors such as climate and vegetation in southern and northern China. Southern China has a relatively humid climate and high forest coverage, where drought exerts a weak limiting effect on fuel load [34]. However, short-term droughts can increase the probability of wildfires by drying out combustible materials [32], suggesting that southern China has a drought-driven fire regime. The northern regions of China experience a relatively dry climate with high evaporation rates [128,129], and drought exerts a strong limiting effect on fuel load compared to southern regions, demonstrating a fuel-limited fire regime.

The probability of forest fire occurrence in the EC, SC, and SWC regions tends to show a decreasing trend in the near and medium term. This is in contrast to a previous study, which indicated that wildfire risk may increase in humid areas [130]. This may be because in our study, the drought characteristics showed a decreasing trend in the near and medium term, and decreasing drought makes the surface fuel wetter, thus reducing the forest fire occurrence probability [131]. In addition, these regions are more densely populated, and the forest fuel treatment measures are often used, such as prescribed burning, fuel reduction. Therefore, the wildfire risk is lower [132]. Moreover, the implementation of strict forest fire suppression policies may also contribute to the reduction of forest fire occurrence probability [133].

The probability of forest fire occurrence in the NC, NEC, and NWC regions mostly showed an increasing trend. Especially in the Northeast region (NEC) under both drought thresholds in the near and medium term. We found that drought characteristics exhibit a predominantly inhibiting effect on the probability of forest fire occurrence in northern China. The increasing trends may be due to decreasing drought conditions, which result in an increase in forest fire occurrence in those regions. Our results were consistent with previous studies [134–136]. The future warmer climatic conditions may lengthen the growing season of plants, and reduced drought increases fuel accumulation, further exacerbating forest fire disturbances [136]. In addition, future climate warming may increase evapotranspiration rates and reduce soil and vegetation moisture, along with the strong spring and summer warming and earlier snowmelt in Northeast China [137], creating conditions with high fire-hazard weather [136,138]. Moreover, longer drought periods [112] and increased variability in rainfall and humidity will also significantly increase wildfire risk [139]. Furthermore, higher vegetation cover in the NEC also increases the probability of forest fire occurrence [136]. Our results highlight the importance of enhancing forest fire monitoring in the NC, NEC, and NWC regions. Specifically, more forest fire monitoring facilities and forest fire fighting resources should be deployed in the NEC.

### 4.4 Limitations and future perspectives

This study developed a forest fire occurrence probability prediction model for China, based on drought characteristics, natural, and anthropogenic variables, potentially excluding other important variables like lightning [140], wind, or human ignition sources beyond population/road density [141]. Although the model demonstrates satisfactory predictive performance (AUC > 0.75), it still has certain limitations. Firstly, the test set contains relatively fewer wildfires compared to the training set, which may not adequately account for the spatial heterogeneity of forest fire occurrence probability. Then, the resolution of SPEI data may not be fine enough to accurately capture localized drought conditions, leading to less accurate predictions for specific areas [142,143]. Finally, we selected the Thornthwaite method to calculate the PET. However, Thornthwaite is a simpler temperature-based empirical method, which may not perform well in all climate regions.

Future studies should focus on (1) using the Penman-Monteith method or more advanced approaches to compute PET; (2) improving the spatial resolution of SPEI data for better prediction of forest fire occurrence probability; and (3) considering a fuller range of influencing factors, especially the thunder and lightning data, to construct prediction models.

## 5 Conclusions

By establishing the geographically weighted logistic regression prediction model for forest fire occurrence probability based on drought characteristics, this study incorporates the spatial non-stationarity of drought characteristics’ influence on forest fire occurrence probability. This approach overcomes the limitations of linear prediction models that solely rely on drought indices and forest fire. We found that the model performed well in its predictions (AUC > 0.75). By comparing the Brier scores, it was found that the models with better predictive performance were those using the SPEI-1 and SPEI-12 timescales. The factors affecting the probability of forest fire occurrence exhibit spatial non-stationarity, and the drought characteristics exhibit a predominantly promoting effect on forest fire occurrence probability in southern China and a predominantly inhibiting effect on forest fire occurrence probability in northern China. Comparison of drought characteristics in future periods reveals that most of the regions in China showed a wetting trend in the near and medium term. We also found that in the future, with climate change, the forest fire occurrence probability in most forest land of northern China (NWC, NC, and NEC), especially in Northeast China (NEC), shows an increasing trend, but a decreasing trend in most forest land of southern China (SC, SWC, and EC). In the near and medium term of the future, due to the accelerated rate of climate change caused by global warming, more forest fire monitoring facilities and forest fire fighting resources need to be deployed in northern China (NC, NEC, and NWC), especially in Northeast China (NEC). Given the relatively frequent occurrence of forest fires in southern China (SC, SWC, and EC), forest fire prevention and control in this region must not be overlooked.

## Supporting information

S1 Text **S1 File.** Data for Fig 3. **S2 File.** Data for Fig 4. **S3 File.** Data for Fig 5. **S4 File.** Data for Fig 6.
(RAR)
